# The genome sequence of the White-pinion Spotted, *Lomographa bimaculata *(Fabricius, 1775)

**DOI:** 10.12688/wellcomeopenres.20841.1

**Published:** 2024-02-29

**Authors:** Douglas Boyes, Inez Januszczak, David C. Lees, University of Oxford and Wytham Woods Genome Acquisition Lab, Darwin Tree of Life Barcoding collective, Wellcome Sanger Institute Tree of Life Management, Samples and Laboratory team, Wellcome Sanger Institute Scientific Operations: Sequencing Operations, Wellcome Sanger Institute Tree of Life Core Informatics team, Tree of Life Core Informatics collective, Darwin Tree of Life Consortium

**Affiliations:** 1 UK Centre for Ecology & Hydrology, Wallingford, England, UK; 2 Natural History Museum, London, England, UK

**Keywords:** Lomographa bimaculata, white-pinioned spotted, genome sequence, chromosomal, Lepidoptera

## Abstract

We present a genome assembly from an individual male *Lomographa bimaculata* (the White-pinion Spotted; Arthropoda; Insecta; Lepidoptera; Geometridae). The genome sequence is 554.7 megabases in span. Most of the assembly is scaffolded into 31 chromosomal pseudomolecules, including the Z sex chromosome. The mitochondrial genome has also been assembled and is 16.66 kilobases in length. Gene annotation of this assembly on Ensembl identified 12,749 protein coding genes.

## Species taxonomy

Eukaryota; Opisthokonta; Metazoa; Eumetazoa; Bilateria; Protostomia; Ecdysozoa; Panarthropoda; Arthropoda; Mandibulata; Pancrustacea; Hexapoda; Insecta; Dicondylia; Pterygota; Neoptera; Endopterygota; Amphiesmenoptera; Lepidoptera; Glossata; Neolepidoptera; Heteroneura; Ditrysia; Obtectomera; Geometroidea; Geometridae; Ennominae; *Lomographa*; *Lomographa bimaculata* (Fabricius, 1775) (NCBI:txid393326).

## Background

*Lomographa bimaculata*, or the White-pinion Spotted, is the type species of the genus *Lomographa*, originally described as *Geometra taminata* [Denis & Schiffermüller], 1775, now a synonym of *L. bimaculata* (Fabricius, 1775). The White-pinion Spotted has an adult wingspan of 22–26 mm (about 13–14 mm forewing length; Waring *et al.*, 2017). The adults are nocturnal. The caterpillars are greenish with a purplish-red dorsal line, but they may vary in colour from a light yellow to a dark red through their various instars ( Buckler, 1897). The larvae feed on hawthorn ( *Crataegus* sp.) and blackthorn ( *Prunus spinosa*), and other Rosaceae shrubs ( Henwood *et al.*, 2020; Leather, 1991), and the moth occurs in woodland open areas with such foodplants. Adults are nocturnal, with white background wings, distinguished by two black inverted triangular costal spots at the top of each convex dotted crossline. They fly from mid-April to mid-July in the UK with a peak in early June ( Randle *et al.*, 2019).

*L. bimaculata* is common in most of the British Isles but very local in southern Scotland and Ireland ( Waring *et al.*, 2017). It can be found in mainly woodland areas over most of Europe although absent in southern Spain and northern Scandinavia. Its distribution extends eastward through Russia and Central Asia to Japan ( GBIF Secretariat, 2023). Unlike many other UK moth species, *L. bimaculata* has been slightly increasing its range, with an annual population change increase over 35 years of 1% ( Conrad *et al.*, 2006), yet from 1970 to 2016 its abundance declined by 23% ( Randle *et al.*, 2019), and has been expanding northwards ( Waring *et al.*, 2017).

Neighbour-joining trees for its DNA barcodes (BOLD, 15/12/2023) reveal that *L. bimaculata* (cluster number, BIN BOLD:AAB9407) has a mean pairwise divergence spanning 0.07% to 0.38% ( *n* = 40). *L. bimaculata* is a mitochondrially very isolated species, with no other *Lomographa* within the top 99 hit on BOLD (over 6.17 % distant to other geometrids). The other British *Lomographa*, *L. temerata* is around 9.8% pairwise divergent to it based on UK DNA barcodes. The species was treated in a multi-locus study by Murillo-Ramos *et al*. (2019), and belongs to the ennomine tribe Baptini, most closely related to the tribe Therini. The genome will be useful to further explore and revise the higher taxonomy of the genus.

We present a chromosomally complete genome sequence for the White-pinion Spotted ( *Lomographa bimaculata*) as part of the Darwin Tree of Life Project. This project is a collaborative effort to sequence all named eukaryotic species in the Atlantic Archipelago, encompassing Britain and Ireland.

## Genome sequence report

**Figure 1.**
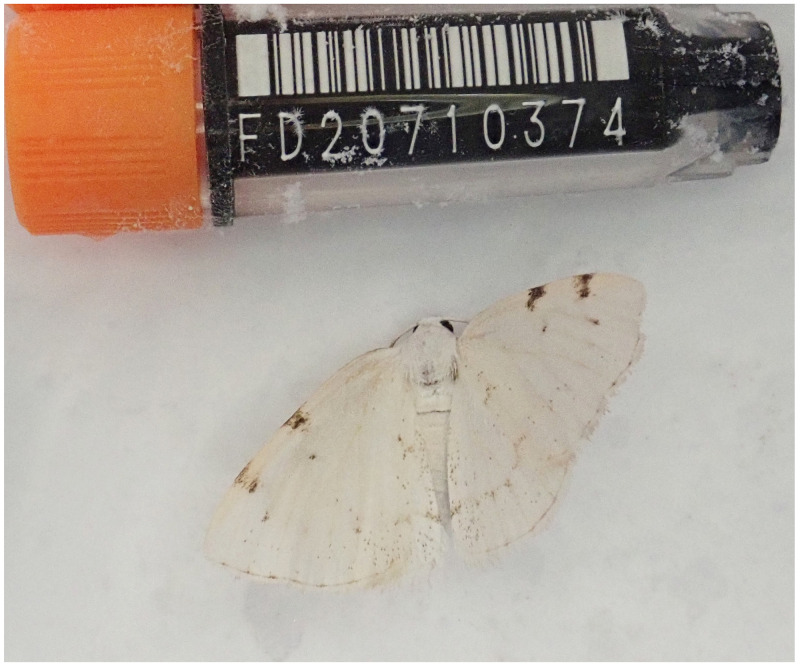
Photograph of the *Lomographa bimaculata* (ilLomBima1) specimen used for genome sequencing.

The genome was sequenced from one male *Lomographa bimaculata* ( Figure 1) collected from Wytham Woods, Oxfordshire, UK (51.77, –1.32). A total of 48-fold coverage in Pacific Biosciences single-molecule HiFi long reads was generated. Primary assembly contigs were scaffolded with chromosome conformation Hi-C data. Manual assembly curation corrected 3 missing joins or mis-joins and removed 1 haplotypic duplications, reducing the scaffold number by 2.94%.

**Figure 2.**
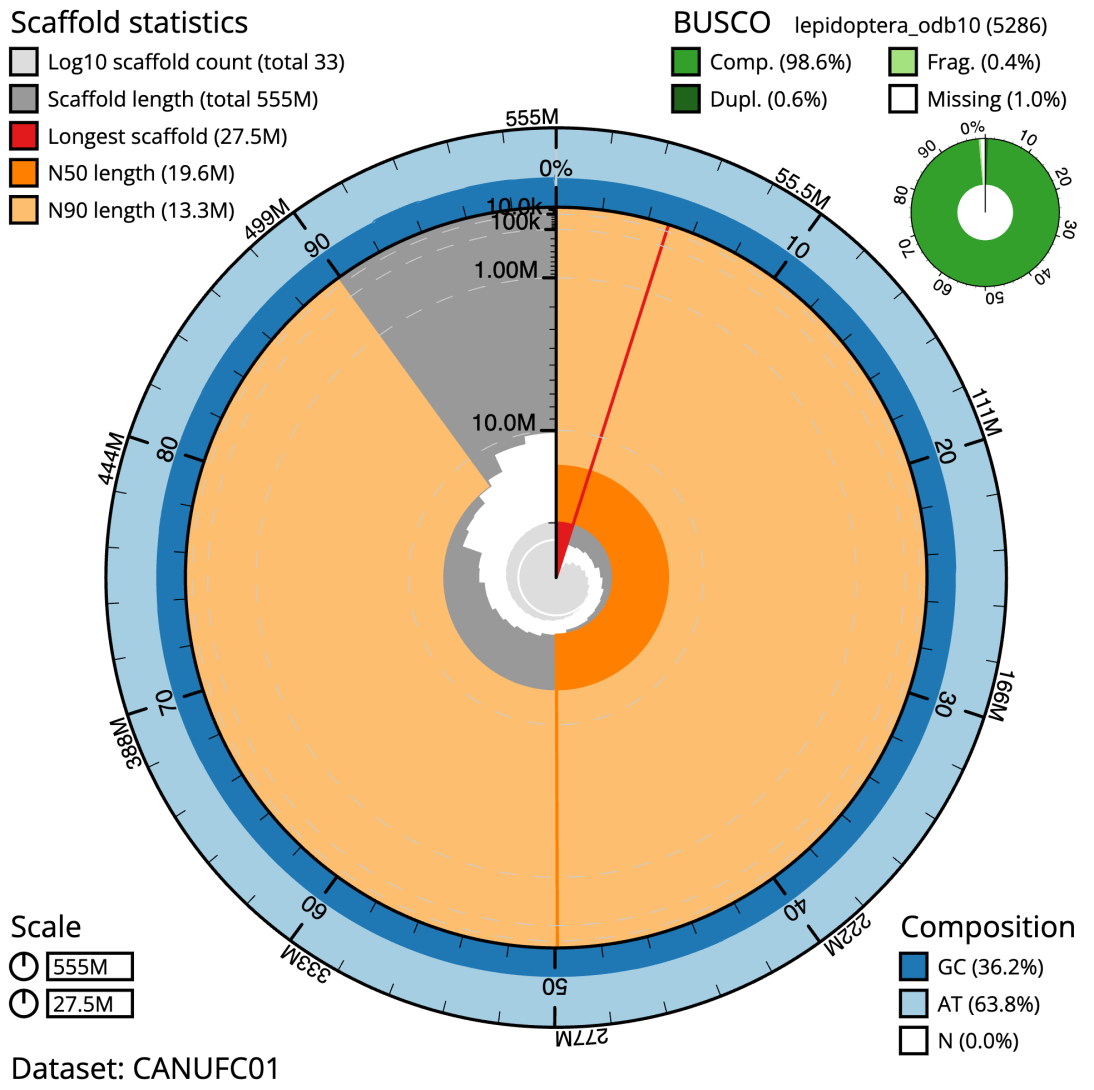
Genome assembly of *Lomographa bimaculata*, ilLomBima1.1: metrics. The BlobToolKit Snailplot shows N50 metrics and BUSCO gene completeness. The main plot is divided into 1,000 size-ordered bins around the circumference with each bin representing 0.1% of the 554,752,746 bp assembly. The distribution of scaffold lengths is shown in dark grey with the plot radius scaled to the longest scaffold present in the assembly (27,520,313 bp, shown in red). Orange and pale-orange arcs show the N50 and N90 scaffold lengths (19,622,098 and 13,309,989 bp), respectively. The pale grey spiral shows the cumulative scaffold count on a log scale with white scale lines showing successive orders of magnitude. The blue and pale-blue area around the outside of the plot shows the distribution of GC, AT and N percentages in the same bins as the inner plot. A summary of complete, fragmented, duplicated and missing BUSCO genes in the lepidoptera_odb10 set is shown in the top right. An interactive version of this figure is available at https://blobtoolkit.genomehubs.org/view/CANUFC01/dataset/CANUFC01/snail.

**Figure 3.**
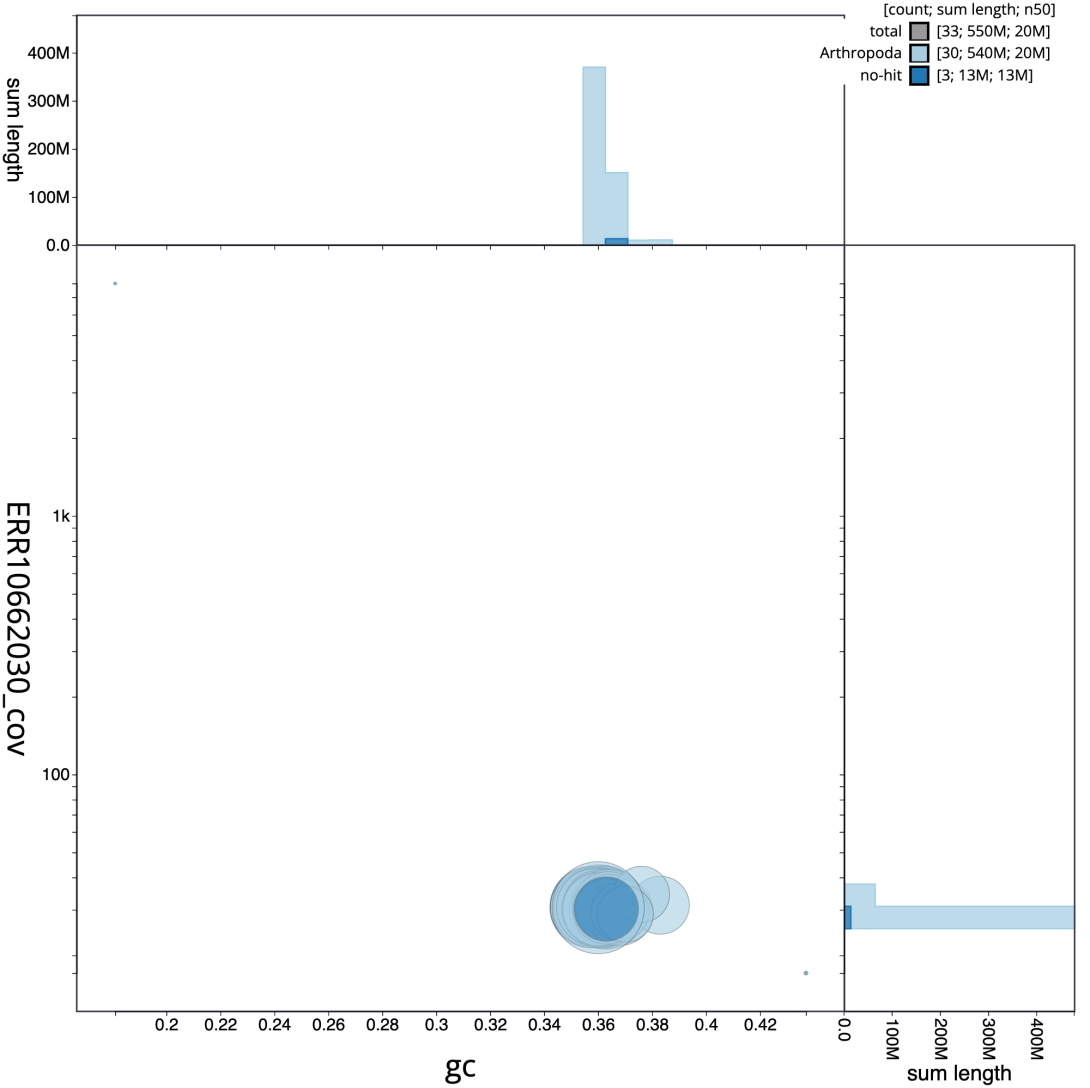
Genome assembly of *Lomographa bimaculata*, ilLomBima1.1: BlobToolKit GC-coverage plot. Scaffolds are coloured by phylum. Circles are sized in proportion to scaffold length. Histograms show the distribution of scaffold length sum along each axis. An interactive version of this figure is available at https://blobtoolkit.genomehubs.org/view/CANUFC01/dataset/CANUFC01/blob.

**Figure 4.**
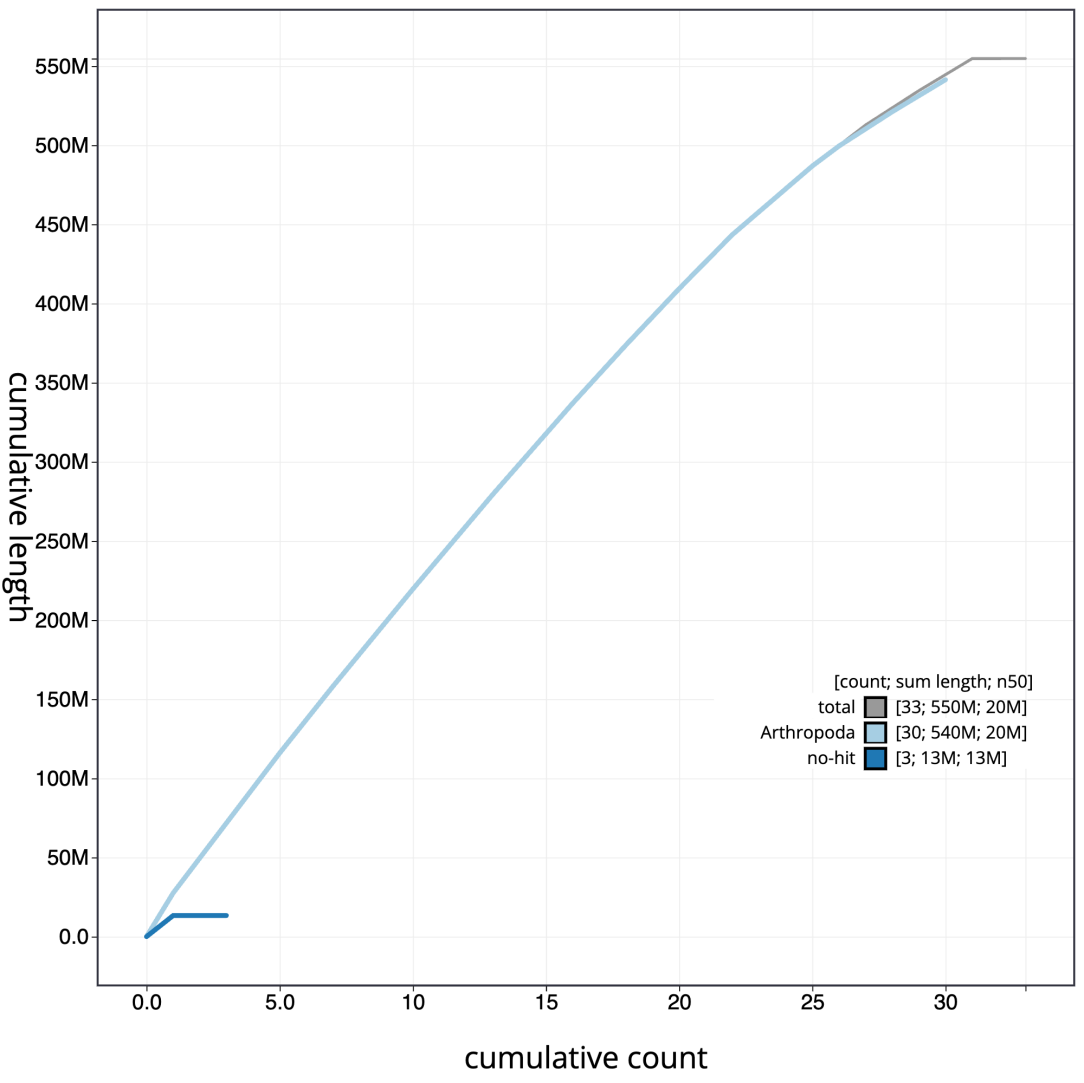
Genome assembly of *Lomographa bimaculata*, ilLomBima1.1: BlobToolKit cumulative sequence plot. The grey line shows cumulative length for all scaffolds. Coloured lines show cumulative lengths of scaffolds assigned to each phylum using the buscogenes taxrule. An interactive version of this figure is available at https://blobtoolkit.genomehubs.org/view/CANUFC01/dataset/CANUFC01/cumulative.

**Figure 5.**
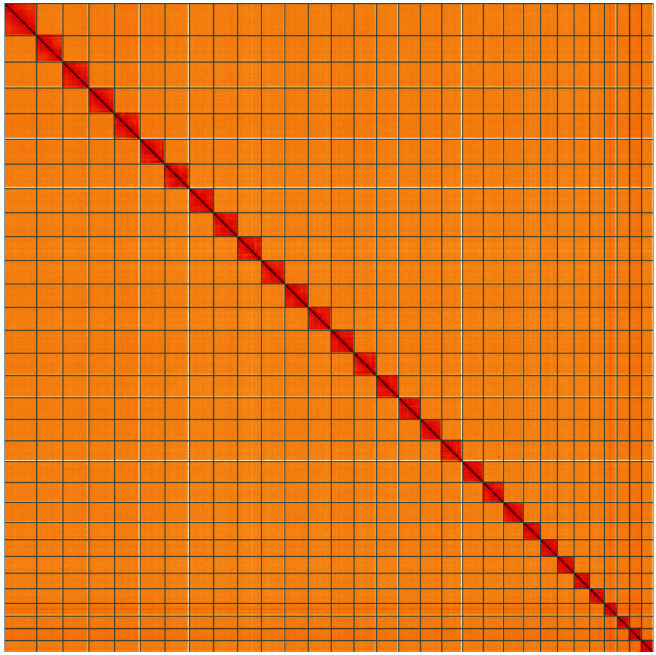
Genome assembly of *Lomographa bimaculata*, ilLomBima1.1: Hi-C contact map of the ilLomBima1.1 assembly, visualised using HiGlass. Chromosomes are shown in order of size from left to right and top to bottom. An interactive version of this figure may be viewed at https://genome-note-higlass.tol.sanger.ac.uk/l/?d=QsVq0YVKSwODyXqtnd55xQ.

The final assembly has a total length of 554.7 Mb in 32 sequence scaffolds with a scaffold N50 of 19.6 Mb ( Table 1). The snailplot in Figure 2 provides a summary of the assembly statistics, while the distribution of assembly scaffolds on GC proportion and coverage is shown in Figure 3. The cumulative assembly plot in Figure 4 shows curves for subsets of scaffolds assigned to different phyla. Most (99.99%) of the assembly sequence was assigned to 31 chromosomal-level scaffolds, representing 30 autosomes and the Z sex chromosome. Chromosome-scale scaffolds confirmed by the Hi-C data are named in order of size ( Figure 5; Table 2). While not fully phased, the assembly deposited is of one haplotype. Contigs corresponding to the second haplotype have also been deposited. The mitochondrial genome was also assembled and can be found as a contig within the multifasta file of the genome submission.

**Table 1.** Genome data for *Lomographa bimaculata*, ilLomBima1.1.

Project accession data		
Assembly identifier	ilLomBima1.1	
Species	*Lomographa bimaculata*	
Specimen	ilLomBima1	
NCBI taxonomy ID	393326	
BioProject	PRJEB58085	
BioSample ID	SAMEA10979145	
Isolate information	ilLomBima1 ilLomBima1	
Assembly metrics *		*Benchmark*
Consensus quality (QV)	68.0	*≥ 50*
*k*-mer completeness	100.0%	*≥ 95%*
BUSCO **	C:98.6%[S:98.0%,D:0.6%], F:0.4%,M:1.0%,n:5,286	*C ≥ 95%*
Percentage of assembly mapped to chromosomes	99.99%	*≥ 95%*
Sex chromosomes	ZZ	*localised homologous pairs*
Organelles	Mitochondrial genome: 16.66 kb	*complete single alleles*
Raw data accessions		
PacificBiosciences SEQUEL II	ERR10662030	
Hi-C Illumina	ERR10659259	
PolyA RNA-Seq Illumina	ERR11242511	
Genome assembly		
Assembly accession	GCA_948107665.1	
*Accession of alternate haplotype*	GCA_948107665.1	
Span (Mb)	554.7	
Number of contigs	68	
Contig N50 length (Mb)	14.2	
Number of scaffolds	32	
Scaffold N50 length (Mb)	19.6	
Longest scaffold (Mb)	27.52	
Genome annotation		
Number of protein-coding genes	12,749	
Number of non-coding genes	1,754	
Number of gene transcripts	22,526	

* Assembly metric benchmarks are adapted from column VGP-2020 of “Table 1: Proposed standards and metrics for defining genome assembly quality” from ( Rhie *et al.*, 2021). ** BUSCO scores based on the lepidoptera_odb10 BUSCO set using version 5.3.2. C = complete [S = single copy, D = duplicated], F = fragmented, M = missing, n = number of orthologues in comparison. A full set of BUSCO scores is available at https://blobtoolkit.genomehubs.org/view/CANUFC01/dataset/CANUFC01/busco.

**Table 2.** Chromosomal pseudomolecules in the genome assembly of *Lomographa bimaculata*, ilLomBima1.

INSDC accession	Chromosome	Length (Mb)	GC%
OX403585.1	1	22.46	36.0
OX403586.1	2	22.23	36.0
OX403587.1	3	21.93	36.0
OX403588.1	4	21.82	36.0
OX403589.1	5	21.24	36.5
OX403590.1	6	20.94	35.5
OX403591.1	7	20.61	36.0
OX403592.1	8	20.46	35.5
OX403593.1	9	20.33	35.5
OX403594.1	10	20.1	36.0
OX403595.1	11	20.07	36.0
OX403596.1	12	19.62	36.0
OX403597.1	13	19.54	36.0
OX403598.1	14	19.14	36.0
OX403599.1	15	18.92	36.0
OX403600.1	16	18.55	36.0
OX403601.1	17	18.26	36.0
OX403602.1	18	17.82	36.5
OX403603.1	19	17.79	36.5
OX403604.1	20	17.16	36.0
OX403605.1	21	17.01	36.5
OX403606.1	22	14.73	36.5
OX403607.1	23	14.36	37.0
OX403608.1	24	14.25	36.5
OX403609.1	25	13.31	36.5
OX403610.1	26	12.61	36.5
OX403611.1	27	10.84	38.5
OX403612.1	28	10.84	37.0
OX403613.1	29	10.13	37.5
OX403614.1	30	10.12	37.0
OX403584.1	Z	27.52	36.0
OX403615.1	MT	0.02	18.0

The estimated Quality Value (QV) of the final assembly is 68.0 with *k*-mer completeness of 100.0%, and the assembly has a BUSCO v5.3.2 completeness of 98.6% (single = 98.0%, duplicated = 0.6%), using the lepidoptera_odb10 reference set ( *n* = 5,286).

Metadata for specimens, barcode results, spectra estimates, sequencing runs, contaminants and pre-curation assembly statistics are given at https://links.tol.sanger.ac.uk/species/393326.

## Genome annotation report

The *Lomographa bimaculata* genome assembly (GCA_948107665.1) was annotated using the Ensembl rapid annotation pipeline ( Table 1; https://rapid.ensembl.org/Lomographa_bimaculata_GCA_948107665.1/Info/Index). The resulting annotation includes 23,526 transcribed mRNAs from 12,749 protein-coding and 1,754 non-coding genes.

## Methods

### Sample acquisition and nucleic acid extraction

A male *Lomographa bimaculata* (specimen ID Ox001884, ToLID ilLomBima1) was collected from Wytham Woods, Oxfordshire (biological vice-county Berkshire), UK (latitude 51.77, longitude –1.32) on 2021-05-28 using a light trap. The specimen was collected and identified by Douglas Boyes (University of Oxford) and preserved on dry ice.

The workflow for high molecular weight (HMW) DNA extraction at the Wellcome Sanger Institute (WSI) includes a sequence of core procedures: sample preparation; sample homogenisation, DNA extraction, fragmentation, and clean-up. In sample preparation, the ilLomBima1 sample was weighed and dissected on dry ice ( Jay *et al.*, 2023). Tissue from the head and thorax was homogenised using a PowerMasher II tissue disruptor ( Denton *et al.*, 2023a).

HMW DNA was extracted in the WSI Scientific Operations core using the Automated MagAttract v2 protocol ( Oatley *et al.*, 2023). The DNA was sheared into an average fragment size of 12–20 kb in a Megaruptor 3 system with speed setting 31 ( Bates *et al.*, 2023). Sheared DNA was purified by solid-phase reversible immobilisation ( Strickland *et al.*, 2023): in brief, the method employs a 1.8X ratio of AMPure PB beads to sample to eliminate shorter fragments and concentrate the DNA. The concentration of the sheared and purified DNA was assessed using a Nanodrop spectrophotometer and Qubit Fluorometer and Qubit dsDNA High Sensitivity Assay kit. Fragment size distribution was evaluated by running the sample on the FemtoPulse system.

RNA was extracted from abdomen tissue of ilLomBima1 in the Tree of Life Laboratory at the WSI using the RNA Extraction: Automated MagMax™ *mir*Vana protocol ( do Amaral *et al.*, 2023). The RNA concentration was assessed using a Nanodrop spectrophotometer and a Qubit Fluorometer using the Qubit RNA Broad-Range Assay kit. Analysis of the integrity of the RNA was done using the Agilent RNA 6000 Pico Kit and Eukaryotic Total RNA assay.

Protocols developed by the WSI Tree of Life laboratory are publicly available on protocols.io ( Denton *et al.*, 2023b).

### Sequencing

Pacific Biosciences HiFi circular consensus DNA sequencing libraries were constructed according to the manufacturers’ instructions. Poly(A) RNA-Seq libraries were constructed using the NEB Ultra II RNA Library Prep kit. DNA and RNA sequencing was performed by the Scientific Operations core at the WSI on Pacific Biosciences SEQUEL II (HiFi) and Illumina NovaSeq 6000 (RNA-Seq) instruments. Hi-C data were also generated from remaining head and thorax tissue of ilLomBima1 using the Arima2 kit and sequenced on the Illumina NovaSeq 6000 instrument.

### Genome assembly, curation and evaluation

Assembly was carried out with Hifiasm ( Cheng *et al.*, 2021) and haplotypic duplication was identified and removed with purge_dups ( Guan *et al.*, 2020). The assembly was then scaffolded with Hi-C data ( Rao *et al.*, 2014) using YaHS ( Zhou *et al.*, 2023). The assembly was checked for contamination and corrected as described previously ( Howe *et al.*, 2021). Manual curation was performed using HiGlass ( Kerpedjiev *et al.*, 2018) and Pretext ( Harry, 2022). The mitochondrial genome was assembled using MitoHiFi ( Uliano-Silva *et al.*, 2023), which runs MitoFinder ( Allio *et al.*, 2020) or MITOS ( Bernt *et al.*, 2013) and uses these annotations to select the final mitochondrial contig and to ensure the general quality of the sequence.

A Hi-C map for the final assembly was produced using bwa-mem2 ( Vasimuddin *et al.*, 2019) in the Cooler file format ( Abdennur & Mirny, 2020). To assess the assembly metrics, the *k*-mer completeness and QV consensus quality values were calculated in Merqury ( Rhie *et al.*, 2020). This work was done using Nextflow ( Di Tommaso *et al.*, 2017) DSL2 pipelines “sanger-tol/readmapping” ( Surana *et al.*, 2023a) and “sanger-tol/genomenote” ( Surana *et al.*, 2023b). The genome was analysed within the BlobToolKit environment ( Challis *et al.*, 2020) and BUSCO scores ( Manni *et al.*, 2021; Simão *et al.*, 2015) were calculated.

Table 3 contains a list of relevant software tool versions and sources.

**Table 3.** Software tools: versions and sources.

Software tool	Version	Source
BlobToolKit	4.2.1	https://github.com/blobtoolkit/blobtoolkit
BUSCO	5.3.2	https://gitlab.com/ezlab/busco
Hifiasm	0.16.1-r375	https://github.com/chhylp123/hifiasm
HiGlass	1.11.6	https://github.com/higlass/higlass
Merqury	MerquryFK	https://github.com/thegenemyers/MERQURY.FK
MitoHiFi	2	https://github.com/marcelauliano/MitoHiFi
PretextView	0.2	https://github.com/wtsi-hpag/PretextView
purge_dups	1.2.3	https://github.com/dfguan/purge_dups
sanger-tol/genomenote	v1.0	https://github.com/sanger-tol/genomenote
sanger-tol/readmapping	1.1.0	https://github.com/sanger-tol/readmapping/tree/1.1.0
YaHS	1.2a	https://github.com/c-zhou/yahs

### Genome annotation

The Ensembl Genebuild annotation system ( Aken *et al.*, 2016) was used to generate annotation for the *Lomographa bimaculata* assembly (GCA_948107665.1). Annotation was created primarily through alignment of transcriptomic data to the genome, with gap filling via protein-to-genome alignments of a select set of proteins from UniProt ( UniProt Consortium, 2019).

### Wellcome Sanger Institute – Legal and Governance

The materials that have contributed to this genome note have been supplied by a Darwin Tree of Life Partner. The submission of materials by a Darwin Tree of Life Partner is subject to the **‘Darwin Tree of Life Project Sampling Code of Practice’**, which can be found in full on the Darwin Tree of Life website here. By agreeing with and signing up to the Sampling Code of Practice, the Darwin Tree of Life Partner agrees they will meet the legal and ethical requirements and standards set out within this document in respect of all samples acquired for, and supplied to, the Darwin Tree of Life Project.

Further, the Wellcome Sanger Institute employs a process whereby due diligence is carried out proportionate to the nature of the materials themselves, and the circumstances under which they have been/are to be collected and provided for use. The purpose of this is to address and mitigate any potential legal and/or ethical implications of receipt and use of the materials as part of the research project, and to ensure that in doing so we align with best practice wherever possible. The overarching areas of consideration are:

•   Ethical review of provenance and sourcing of the material

•   Legality of collection, transfer and use (national and international)

Each transfer of samples is further undertaken according to a Research Collaboration Agreement or Material Transfer Agreement entered into by the Darwin Tree of Life Partner, Genome Research Limited (operating as the Wellcome Sanger Institute), and in some circumstances other Darwin Tree of Life collaborators.

## Data Availability

European Nucleotide Archive: *Lomographa bimaculata* (white-pinion spotted). Accession number PRJEB58085; https://identifiers.org/ena.embl/PRJEB58085 ( Wellcome Sanger Institute, 2023). The genome sequence is released openly for reuse. The *Lomographa bimaculata* genome sequencing initiative is part of the Darwin Tree of Life (DToL) project. All raw sequence data and the assembly have been deposited in INSDC databases. Raw data and assembly accession identifiers are reported in Table 1.

## Author information

Members of the University of Oxford and Wytham Woods Genome Acquisition Lab are listed here: https://doi.org/10.5281/zenodo.7125292.

Members of the Darwin Tree of Life Barcoding collective are listed here: https://doi.org/10.5281/zenodo.4893703.

Members of the Wellcome Sanger Institute Tree of Life Management, Samples and Laboratory team are listed here: https://doi.org/10.5281/zenodo.10066175.

Members of Wellcome Sanger Institute Scientific Operations: Sequencing Operations are listed here: https://doi.org/10.5281/zenodo.10043364.

Members of the Wellcome Sanger Institute Tree of Life Core Informatics team are listed here: https://doi.org/10.5281/zenodo.10066637.

Members of the Tree of Life Core Informatics collective are listed here: https://doi.org/10.5281/zenodo.5013541.

Members of the Darwin Tree of Life Consortium are listed here: https://doi.org/10.5281/zenodo.4783558.
