# Supplementary material for: The genome sequence of the White-pinion Spotted, Lomographa bimaculata (Fabricius, 1775)
Source: Wellcome Open Res. 2024 Feb 29;9:96. [Version 1] doi: 10.12688/wellcomeopenres.20841.1 (PMC11384207; doi:10.12688/wellcomeopenres.20841.1)
Supplement: Peer review history

# Reviewer report: Reviewer response for version 1

**DOI:** 10.21956/wellcomeopenres.23060.r90111

**Published:** 2024-09-09

**Authors:** Antonio Ortiz

**Affiliations:** 1 University of Murcia, Murcia, Spain

Boyes, Januszczak & Lees describes the genome assembly of *Lomographa bimaculata *and provides important genomic information about its size (near 555 Mb) in 31 chromosomes. A new genome sequenced from the moth family Geometridae is added to others species of the family as *Thera britannica *(Turner, 1925) by Boyes & Holland (2023), *Eupithecia vulgata* (Haworth, 1809) by Boyes & Mulley (2023), *Eupithecia insigniata *(Hübner, 1790) by Holland (2023), *Eulithis prunata* (Linnaeus, 1758) by Boyes & Holland (2023) all published in Wellcome Open Research (WOR). This assembly provides valuable information for the genomics of Lepidoptera and for further phylogenetic research in the evolutionary pattern in Lepidoptera.

 The methods for the sequencing and assembly used are appropriate and technically supported.

Are sufficient details of methods and materials provided to allow replication by others?

Yes

Is the rationale for creating the dataset(s) clearly described?

Yes

Are the datasets clearly presented in a useable and accessible format?

Yes

Are the protocols appropriate and is the work technically sound?

Yes

Reviewer Expertise:

Systematics and Evolution of Lepidoptera and other insects

I confirm that I have read this submission and believe that I have an appropriate level of expertise to confirm that it is of an acceptable scientific standard.

# Reviewer report: Reviewer response for version 1

**DOI:** 10.21956/wellcomeopenres.23060.r86951

**Published:** 2024-07-16

**Authors:** Savarimuthu Ignacimuthu, Sivasankaran Kuppusamy

**Affiliations:** 1 St. Xavier's College, Interdisciplinary Research Centre in Biology, Xavier Research Foundation, Manonmaniam Sundaranar University, Thirunelveli, TN, India; 2 Entomology Research Institute, Loyola College, Chennai, Tamil Nadu, India

The authors have assembled and annotated of *Lomographa bimaculata* (Fabricius, 1775) genomic sequence. They sequenced about 554.7 mega bases of the assembled genome using 32 scaffolds. Total of 12, 749 protein coding genes, 1,754 non-coding genes and the largest number of gene transcripts (22,526) have been observed through genome annotation. Standard techniques have been employed to isolate DNA, and the relevant software has been used for sequence assembly and annotation.

**Comments on the manuscript**

1. The authors have constantly given the complete form of the genus name *Lomographa bimaculata’s* in the article. The genus name may be presented in full the first time, and then in abbreviated form later on, such as *L. bimaculata.*
* *
2. The first paragraph of the background the third sentence “The adults are nocturnal” may be deleted. Because the same meaning was provided in the penultimate sentence of the first paragraph.
3. In the fourth sentence of the first paragraph the last line authors have given “foodplants”. It’s not a single word. It can be separated i.e., food plants.
4. The mitochondrial genome was annotated but the exact number of mitochondrial genome sequence lengths was not mentioned in the text. The mitogenome length can be given in the text.
5. Table number and title for table 3 was given in between the text in page number 9. I think it is wrongly placed. The table number and title can be deleted or replaced. 
  Above all, I confirm that the manuscript meets the necessary scientific standard and is suitable for indexing.

Are sufficient details of methods and materials provided to allow replication by others?

Yes

Is the rationale for creating the dataset(s) clearly described?

Yes

Are the datasets clearly presented in a useable and accessible format?

Yes

Are the protocols appropriate and is the work technically sound?

Yes

Reviewer Expertise:

Molecular Biology

We confirm that we have read this submission and believe that we have an appropriate level of expertise to confirm that it is of an acceptable scientific standard.
